# Photocatalytic Arylation of Alkenes, Alkynes and Enones with Diazonium Salts

**DOI:** 10.1002/open.201200011

**Published:** 2012-04-27

**Authors:** Peter Schroll, Durga Prasad Hari, Burkhard König

**Affiliations:** a Department of Chemistry and Pharmacy, Universität Regensburg, Universitätsstr. 3193040 Regensburg (Germany) E-mail: burkhard.koenig@chemie.uni-regensburg.de

**Keywords:** arylation reactions, diazonium salts, photocatalysis, radicals, visible light

Carbon–carbon bond formation by sp^2^–sp^2^ or sp^2^–sp cross-coupling is a key transformation in organic synthesis.[1] Many methods, typically involving transition metal catalysis, are known, and the recent recognition of Richard F. Heck, Ei-ichi Negishi and Akira Suzuki by the Royal Swedish Academy of Sciences (Stockholm, Sweden) when they were awarded the Nobel Prize in Chemistry (2010) underlines the importance of metal-catalyzed cross-coupling reactions.[2] However, long before the triumph of the palladium-catalyzed cross-coupling reaction, such as the Heck (1972) and Sonogashira (1975) reactions,[3] methods for arylation of alkenes and alkynes were known. The Meerwein arylation, developed in 1939, is a copper-catalyzed coupling of an aryl diazonium salt with unsaturated compounds.[4] Even earlier, in 1896, an intramolecular variant of this reaction was reported, today known as the Pschorr reaction.[5] A radical mechanism is discussed for both cases by reversible oxidation of copper(I) to copper(II). However, several drawbacks have prevented the broader application of these reactions in organic synthesis: the reaction yields are typically low (20–40 %), high catalyst loadings are required (15–20 mol %), and side products are formed under the aqueous reaction conditions (Scheme 1).

**Scheme 1:**
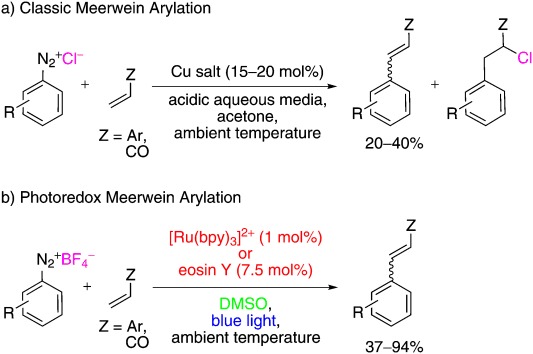
a) Classic Meerwein arylation protocol and b) the related improved photoredox process.

In addition to the reduction of aryl diazonium salts by copper(I) cations, several other methods exist giving access to aryl radicals. Amongst others, aryl radicals can be obtained by photoinduced electron transfer.[6] Organometallic photocatalysts such as 2,2′-bipyridine (bpy)-containing ruthenium complexes (e.g., [Ru(bpy)_3_]^2+^) are known to undergo one-electron transfer reactions.[7] Visible light-induced photoredox catalysis offers the possibility of initiating organic transformations with high selectivities under mild conditions, as demonstrated by MacMillan, Yoon, Stephenson and many others.[8] Current reports describe the photocatalytic formation of carbon–carbon or carbon–heteroatom bonds.[9–12] Recently, visible-light photocatalysis has entered the field of palladium-catalyzed cross-coupling reactions. In 2007, Akita reported the acceleration of copper-free Sonogashira-type reactions by adding a photocatalyst.[13] Sanford et al. reported a merger of palladium-catalyzed C–H functionalization and visible-light photocatalysis. In their approach, aryl radicals are obtained from the photocatalytic reduction of aryl diazonium salts by the aid of [Ru(bpy)_3_]^2+^ and, subsequently, used in palladium-catalyzed C–H arylation reactions.[14] Direct C–H arylation of heteroarenes with aryl diazonium salts was achieved using eosin Y and visible light.[15]

[Ru(bpy)_3_]^2+^ is the catalyst of choice for many photoredox reactions due to its unique photochemical properties: absorption of blue light (*λ*_max_=452 nm), high chemical stability, long lifetime of the photoexcited state, and high quantum yield of its formation.[16] The catalyst is able to reduce aryl diazonium salts, such as *para*-bromophenyldiazonium tetrafluoroborate (**1 f**, *E*_1/2red_=+0.02 V), from the excited state (*E*_1/2ox_=−0.76 V at 293 K) and is therefore able to photochemically form highly reactive aryl radicals (**4)** that can subsequently be trapped by unsaturated compounds (**2**; Scheme 2).[17] Combining the fields of photoredox catalysis and cross-coupling reactions, we report the intermolecular visible-light-mediated arylation of unsaturated compounds catalyzed by [Ru(bpy)_3_]^2+^ or eosin Y as photocatalysts. The process is atom economic and efficient and therefore suitable to improve the classic Meerwein arylation protocol significantly.

The reaction of phenyldiazonium tetrafluoroborate (**1 a**) with styrene (**2 a**) in the presence of [Ru(bpy)_3_]^2+^ under inert atmosphere and irradiation with a blue high-power light-emitting diode (LED, *λ*_max_=455±15 nm, *P*=3 W) at ambient temperature gave stilbene (**3 a**), which is the formal substitution product of a vinylic hydrogen atom by the aryl residue of the diazonium salt. This result is in contrast to the recently reported photocatalytic radical addition reactions of alkyl halides to olefins.[18] Monitoring of the reaction kinetics revealed that the *trans* isomer is initially formed as the major product, but then partially isomerizes to the *cis* isomer upon irradiation (for kinetic data, see the Supporting Information).

In the absence of the photocatalyst or without light, no coupling product is obtained. The use of polar aprotic solvents, such as *N*,*N*-dimethylformamide (DMF) or dimethyl sulfoxide (DMSO) and a fivefold excess of styrene (**2 a**) gave the best results (Table 1). Higher concentrations of **2 a** result in the formation of oligomers and polymers, while at lower concentrations, the lifetime of the aryl radical is too short for the diffusion-controlled reaction with the alkene to occur. Several photocatalysts were screened, but perylene bisimide and rose bengal gave only low yields. Eosin Y (5 mol %) gave a moderate yield of 44 %, while [Ru(bpy)_3_]^2+^ (1 mol %) afforded the product in up to 87 % yield. Higher catalyst loadings of [Ru(bpy)_3_]^2+^ decreased the yield (cf. Entries 9, 10 and 14, Table 1).

**Table 1:** Optimization of reaction conditions[a]

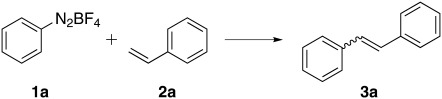						
Entry	Catalyst (mol %)[b]		Styrene [equiv]	*λ* [nm][c]	Solvent	Yield [%][d]
1	–		10	455	DMSO	–
2	[Ru(bpy)_3_]^2+^	(1)	10	–	DMSO	–
3	[Ru(bpy)_3_]^2+^	(1)	10	455	CHCl_3_	–
4	[Ru(bpy)_3_]^2+^	(1)	10	455	THF	25
5	[Ru(bpy)_3_]^2+^	(1)	10	455	DMF	55
6	[Ru(bpy)_3_]^2+^	(1)	10	455	DMSO	62
7	perylene bisimide[e]	(5)	5	520	DMF	3
8	rose bengal	(5)	5	520	DMSO	11
9	eosin Y	(5)	5	520	DMSO	44
10	[Ru(bpy)_3_]^2+^	(1)	5	455	DMSO	87
11	[Ru(bpy)_3_]^2+^	(1)	1	455	DMSO	67
12	[Ru(bpy)_3_]^2+^	(1)	2	455	DMSO	71
13	[Ru(bpy)_3_]^2+^	(0.5)	5	455	DMSO	77
14	[Ru(bpy)_3_]^2+^	(5)	5	455	DMSO	64

[a] *Reagents and conditions*: aryl diazonium salt (0.2 mmol), styrene (0.2–2.0 mmol, 0.02-0.23 mL), photocatalyst (0.5–5 mol %), solvent (0.77–0.98 mL), inert atmosphere, visible light, 20 °C, 2 h.

[b] Amount relative to the amount of diazonium salt.

[c] High power LED (*λ*_max_=455±15 nm, *P*=3 W or *λ*_max_=520±15 nm, *P*=1 W).

[d] Yields were determined by integration of the peaks in the gas chromatogram and are the sum of the *cis* and *trans* isomers.

[e] Catalyst: *N*,*N*′-di(2-hexyl)heptyl-perylene-3,4,9,10-tetracarboxylic bisimide; not soluble in DMSO.

The scope of the reaction was explored using a set of substituted aryl diazonium salts (**1**) and unsaturated compounds (**2**) under optimized reaction conditions: [Ru(bpy)_3_]^2+^ as the photocatalyst with a loading of 1 mol %, DMSO as the solvent, a fivefold excess of olefin (**2**), a nitrogen atmosphere, blue-light irradiation, ambient temperature, and a two-hour reaction time. The use of boron tetrafluoride (BF_4_^−^) as a non-nucleophilic counter ion avoids the formation of addition products (for further details, see the Supporting Information).

A range of different substituted aryl diazonium salts was examined in the arylation of styrene, including electron-withdrawing and donating groups (Table 2). The coupling products **3 a**–**f** were obtained in good to excellent yields of 66–94 %, which was attributed to the appearance of an intermediary formed benzylic radical. Direct sunlight is sufficient to drive the reaction. The yields under these conditions—radiation angle of 37° on the roof of our institute at 48° 59’ N, 12° 6’ E at noon on October 1st, 2011—are similar to those of defined laboratory conditions (Table 2). The coupling of aryl diazonium salts **1 a**–**f** also proceeds under metal-free conditions using eosin Y with a higher catalyst loading of 7.5 mol % and green light (*λ*_max_=520±15 nm). Substituted stilbenes **3 a**–**f** are obtained in 51–80 % yield as *trans* isomers. Several functional groups including ether, alkyl, nitro and halide groups are tolerated in this reaction, but aryl diazonium salts derived from aminophenols lead to decomposition of the starting material. Carbon–halide bonds remain untouched providing access to halogen-substituted stilbenes in one step, which can be further functionalized (Entries 5 and 6, Table 2). One benefit of this method is its atom economy: all atoms, with the exception of molecular nitrogen, the counter ion and a proton, appear in the product molecule.

**Table 2:** Scope of aryl diazonium salts[a]

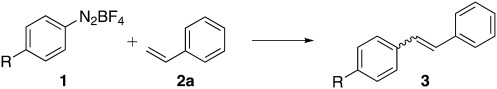						
Entry	Substrate		Product		*T* [°C]	Yield [%][b]
1	**1 a**	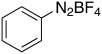	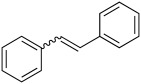	**3a**	20	87
					20	55[d]
2	**1 b**	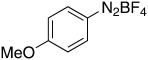	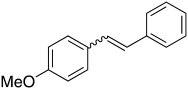	**3 b**	20	83
					37	64[c]
					20	80[d]
3	**1 c**	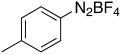	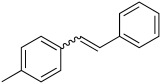	**3 c**	20	68
					37	65[c]
					20	70[d]
4	**1 d**	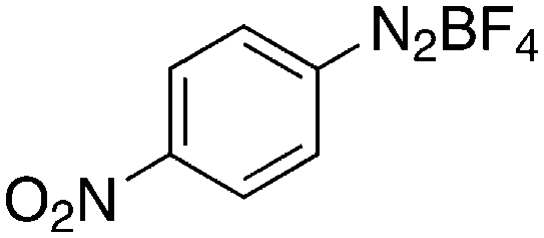	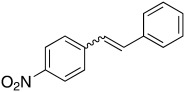	**3 d**	20	66
					37	58[c]
					20	52[d]
5	**1 e**	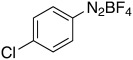	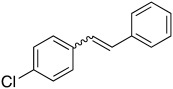	**3 e**	20	72
					37	39[c]
					20	51[d]
6	**1 f**	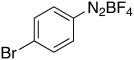	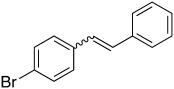	**3 f**	20	94
					20	66[d]

[a] *Reagents and conditions*: aryl diazonium salt (0.2 mmol), styrene (1.0 mmol, 0.12 mL), [Ru(bpy)_3_]^2+^ (1 mol %), DMSO (0.88 mL), 455 nm LED or sunlight, 20 °C, 2 h.

[b] Yields were determined by integration of the peaks in the gas chromatogram and are the sum of the *cis* and *trans* isomers.

[c] Irradiation with sunlight.

[d] Catalyst: eosin Y (7.5 mol %); irradiation with a green LED (*λ*_max_=520±15 nm, *P*=1 W).

Among several unsaturated compounds, styrenes bearing functional groups at the aromatic ring or the double bond were effectively coupled (Table 3). In cases where cinnamic acid (**2 d**) or β-nitrostyrene (**2 e**) was used as the reactant, the coupling was accompanied by defunctionalization leading to the loss of CO_2_ or NO_2_, respectively. Unlike the styrenes, phenylacetylene undergoes sp^2^–sp coupling to form diphenylacetylene in moderate yields, which could be attributed to the reduced reactivity of the triple bond compared with styrene (Entry 3, Table 3). Moreover, enones such as *para*-benzoquinone (**2 i**) or coumarin (**2 k**) were arylated in excellent yields showing that this method is not restricted to benzylic substrates (Entries 4 and 5, Table 3).

**Table 3:** Scope of unsaturated compounds[a]

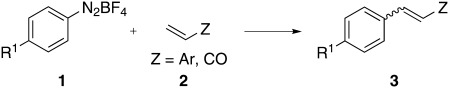							
Entry	R^1^	R^2^	Substrate		Product		Yield [%][b]
1	H H H	MeO Me Br	**2 b**	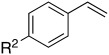	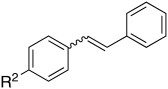	**3 b**	73
			**2 c**			**3 c**	64
			**2 f**			**3 f**	63
2	H H	COOH NO_2_	**2 d**	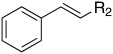	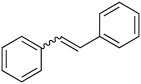	**3 a**	49
			**2 e**			**3 a**	37
3[c]	MeO Cl	– –	**2 g**	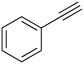	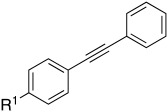	**3 g**	47
			**2 g**			**3 h**	48
4	H	–	**2 i**	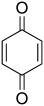	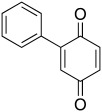	**3 i**	89[d]
5	H	–	**2 k**	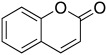	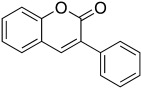	**3 k**	63[d]

[a] *Reagents and conditions*: aryl diazonium salt (0.2 mmol), unsaturated compound (1.0 mmol), [Ru(bpy)_3_]^2+^ (1 mol %), DMSO (1.0 mL), 455 nm LED, 20 °C, 2 h.

[b] Yields were determined by integration of the peaks in the gas chromatogram and are the sum of the *cis* and *trans* isomers.

[c] Catalyst: 2-phenylpyridine (ppy)-containing iridium complex, *fac*-Ir(ppy)_3_ (2 mol %), *λ*_irr_=400±10 nm.

[d] Isolated yield.

A radical pathway including one-electron oxidation and reduction steps is likely for the photoredox arylation. (2,2,6,6-Tetramethylpiperidin-1-yl)oxyl (TEMPO) adducts **7** and **8** were obtained irradiating the reaction mixture and TEMPO, which indicates the presence of aryl radical intermediates **4** and **5**. In methanol, addition product **9** is formed, suggesting the presence of a carbenium ion intermediate (Scheme 2).

**Scheme 2:**
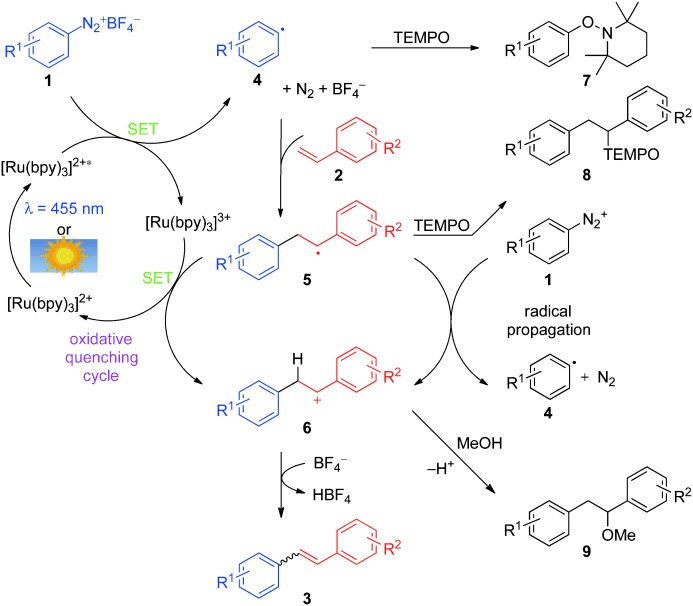
Proposed mechanism for the photoredox arylation of unsaturated compounds using diazonium salts.

Taking the results of these experiments into account, a mechanistic model for the arylation of unsaturated compounds using the oxidative quenching cycle of [Ru(bpy)_3_]^2+^ is proposed (Scheme 2). After excitation of the metal catalyst with blue light, an electron is transferred to diazonium salt **1** and aryl radical **4** is generated upon loss of dinitrogen. The attack of **4** to the double bond of styrene **2** gives benzylic radical **5**, which is oxidized to a carbenium ion (**6**) either by redonating an electron to the oxidized photocatalyst and simultaneously closing the catalytic cycle, or by transferring an electron to another diazonium salt molecule (**1**) initiating a radical chain mechanism. Product **3** is formed after deprotonation.

In conclusion, we have developed an efficient visible-light-mediated arylation of unsaturated compounds by photoredox catalysis. The procedure is experimentally simple and characterized by high yields, low catalyst loadings and mild conditions using sunlight and ambient temperature. The reaction scope comprises a range of different substituted aryl diazonium salts and tolerates a variety of functional groups including aryl halides. Unsaturated compounds such as alkenes, alkynes and enones are effectively coupled. The photoredox procedure improves the classic Meerwein arylation protocol significantly to make it more applicable to organic synthesis.

## Experimental Section

**General procedure**: Ru(bpy)_3_Cl_2_⋅6H_2_O (1 mol %, 1.5 mg), aryl diazonium tetrafluoroborate **1** (1 equiv, 0.2 mmol), unsaturated compound **2** (5 equiv, 1.0 mmol) and dry DMSO (1 mL) were added to a 5-mL reaction vessel equipped with a magnetic stirring bar. The mixture was degassed using the “freeze–pump–thaw” technique (3×) and irradiated with a blue high-power LED (*λ*=455±15 nm) at 20 °C for 2 h. The yield was determined using gas chromatography or by isolation of the product using preparative thin-layer chromatography.

## Supplementary material

Detailed facts of importance to specialist readers are published as ”Supporting Information”. Such documents are peer-reviewed, but not copy-edited or typeset. They are made available as submitted by the authors.
