# Supplementary material for: Photocatalytic Arylation of Alkenes, Alkynes and Enones with Diazonium Salts
Source: ChemistryOpen. 2012 Apr 27;1(3):130–3. doi: 10.1002/open.201200011 (PMC3922450; doi:10.1002/open.201200011)
Supplement: Supplementary file 1 [file open0001-0130-SD1.pdf]

ChemistryOPEN

Supporting Information

© Copyright Wiley-VCH Verlag GmbH & Co. KGaA, 69451 Weinheim, 2012

Photocatalytic Arylation of Alkenes, Alkynes and Enones with Diazonium Salts

Peter Schroll, Durga Prasad Hari, and Burkhard König*[a]

open_201200011_sm_miscellaneous_information.pdf

1. General Methods and Instruments

Irradiation Sources

High Power LEDs of different colors were used for irradiation of the reaction mixtures: Philips LUXEON® Rebel (purple, λ_{max} = 400 ± 10 nm, 1000 mA, 1.2 W), Philips LUXEON® Rebel LXML-TRo1-0225 (blue, λ_{max} = 455 ± 15 nm, 700 mA, 3.0 W) and Philips LUXEON® Rebel (green, λ = 520 ± 15 nm, 145 lm @700mA, 1.0 W).

NMR-Spectroscopy

NMR-spectroscopic measurements were recorded on a Bruker Avance 300 (^{1}H: 300 MHz, ^{13}C: 75 MHz, T = 295 K) and a Bruker Avance 400 (^{1}H: 400 MHz, ^{13}C: 100 MHz, T = 295 K) spectrometer. The chemical shifts are reported in δ [ppm] relative to internal standards (solvent residual peak). The spectra were analyzed by first order, the coupling constants *J* are given in Hertz [Hz].

Characterization of the signals: chemical shift (ppm), multiplicity (s = singlet, d = doublet, t = triplet, q = quartet, quint = quintet, dd = doublet of doublets, dt = doublet of triplets, tt = triplet of triplets, m = multiplet, br. s. = broad singlet), and coupling constants (Hz).

Integration is determined as the relative number of protons. Error of reported values: chemical shift: 0.01 ppm for ^{1}H-NMR, 0.1 ppm for ^{13}C-NMR and 0.1 Hz for coupling constants. The solvent used is reported for each spectrum.

Gas Chromatography

The measurements were done on a GC 7890 from Agilent Technologies. Injector temperature (splitinjection: 40:1 split) was 250°C, detection temperature was 300°C (FID). A capillary column Varian Factor Four VF-5MS / 30 m x 0.25 mm / 0.2 µm film was used. Helium was utilized as carrier gas with a low rate of 1 mL / min. Agilent ChemStation Rev.B.04.02. (96) was used as the software for data acquisition and evaluation.

GC measurements were made and investigated via integration of the signals obtained. The GC oven temperature program adjustment was the follows: the initial temperature of 40°C was kept

for 3 minutes. Then the temperature increased constantly at a rate of 15°C / min for 16 minutes. The final temperature was 280°C. This temperature was kept for 5 minutes.

Solvents and Substrates

Commercial reagents and starting materials were purchased from Aldrich, Fluka, VWR or Acros and used without further purification. Solvents were used as p.a. grade or dried and distilled as described in common procedures.[1]

Preparative Thin-Layer Chromatography

All reactions were monitored by thin-layer chromatography using Merck silica gel plates 60 F254. Visualization was accomplished with UV light ($\lambda = 254$ nm or $\lambda = 366$ nm) or staining with appropriate stains (anisaldehyde or orthophosphomolybdic acid). Preparative thin-layer chromatography was performed using silica gel 60 F254 of particle size 40−63 µm on glass plates 20 x 20 cm.

2. General Procedures

General procedure for the preparation of aryl diazonium tetrafluoroborates 1[2]

The appropriate aniline (1 eq., 10 mmol) was dissolved in a mixture of distilled water (4 mL) and hydrofluoroboric acid (32%, 1 eq., 10 mmol, 5.3 mL). After cooling the reaction mixture to 0°C sodium nitrite (1 eq., 10 mmol, 0.69 g) in water (1.5 mL) was added dropwise. The resulting mixture was stirred for two hours. A thick precipitate was formed, collected by filtration and re-dissolved in a minimum of acetone. The diazonium tetrafluoroborate was precipitated by the addition of diethyl ether. Further purification was achieved by repeating the procedure of dissolving the solid in acetone and precipitating the product by addition of diethyl ether, until the product was obtained as a white solid. The aryl diazonium salt **1** was filtered, washed three times with diethyl ether and dried in vacuo.

General procedure for photocatalytic arylation reactions

A 5 mL reaction vessel with a magnetic stirring bar was equipped with $Ru(bpy)_3Cl_2{\cdot}6H_2O$ (1 mol%, 1.5 mg), aryl diazonium tetraflouroborate **1** (1 eq., 0.2 mmol), unsaturated compound **2** (5 eq., 1.0 mmol) and dry DMSO (1 mL). The mixture was degassed by "freeze-pump-thaw" technique (three cycles) and irradiated with blue high power LEDs ($\lambda_{max} = 455 \pm 15$ nm, P = 3 W) for two hours at a temperature of 20°C. The yield of the reaction was determined via pathway a) or b).

a) GC-analysis:

   The reaction mixture was filtered, and a sample for GC analysis was prepared by diluting the reaction mixture (500 µL) with DMSO (250 µL) and a stock solution of naphthalene (250 µL, β = 15 mg/mL in DMSO) as the standard. The yield was determined by integration of the peaks in the gas chromatogram.

b) Isolation and purification:

   The reaction mixture was diluted with water (4 ml) and extracted with diethyl ether (3 x 5 mL). The combined organic layers were concentrated in vacuum. Traces of water were removed by lyophilization. Purification of the crude product was achieved by preparative thin-layer chromatography on silica gel 60 F254 using a mixture of petroleum ether and ethyl acetate as eluent.

3. Kinetics

Monitoring of the reaction kinetics for the $[Ru(bpy)_3]^{2+}$-mediated arylation of styrene showed that initially the *trans* isomer is formed as the major product. A partial isomerization to the *cis* isomer was observed upon irradiation with blue light (λ_{max} = 455 ± 15 nm) leading to a product distribution of 5:2 (*trans*:*cis*) after two hours of reaction time. Further irradiation does not lead to an increase of product formation. Nitrogen evolution starts as soon as the light is switched on and ends immediately after removal of the radiation source (Figure 1). In case of eosin Y as the photocatalyst, the yields are generally lower and the *trans* isomer is formed exclusively. The nonappearance of photo isomerization can be explained by the lower energy of the green light (high power LED, λ_{max} = 520 ± 15 nm) used to excite eosin Y.

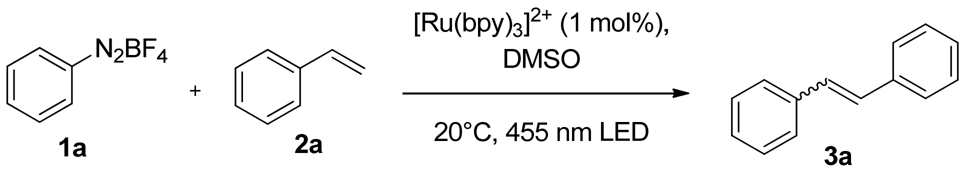

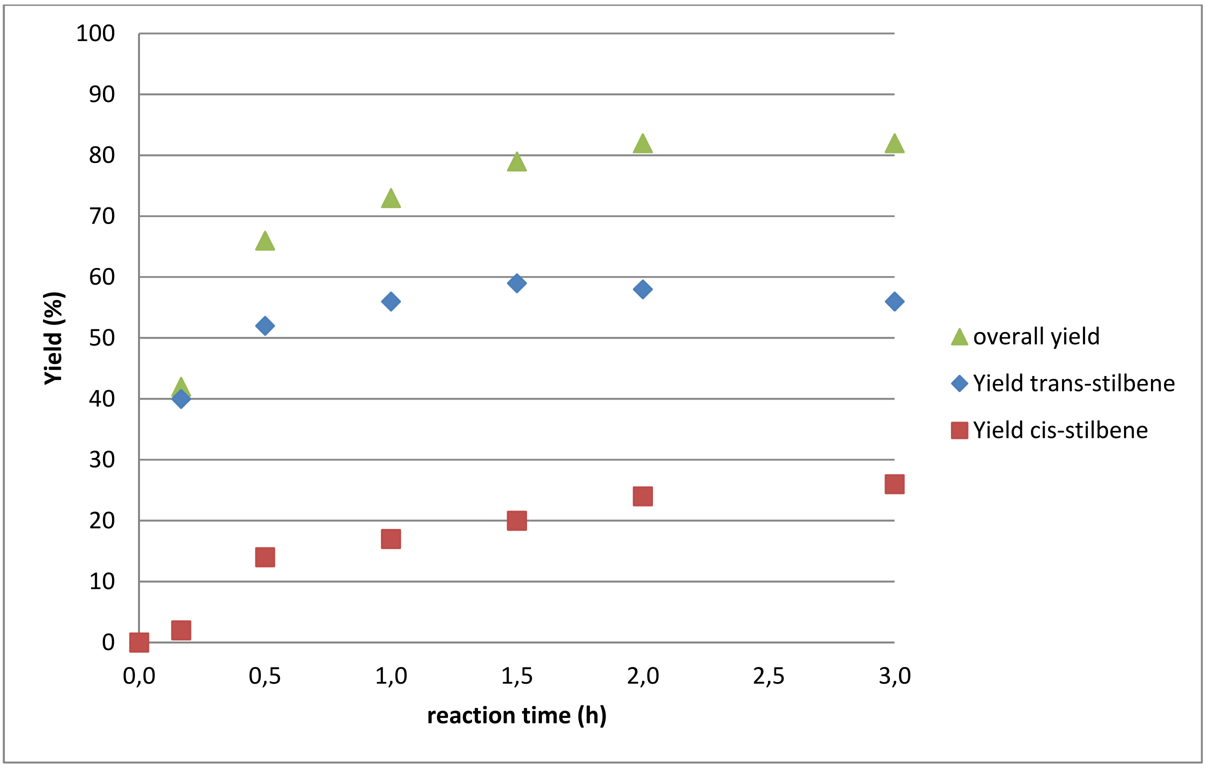

Figure 1. Kinetics of the photocatalytic arylation of styrene.

4. Mechanistic Model and Trapping Experiments

Based on the results of our coupling and trapping experiments as well as the consideration of literature-known data, a mechanistic model for the visible light-mediated arylation of unsaturated compounds catalyzed by $[Ru(bpy)_3]^{2+}$ was developed (Scheme 1).[3,4,5,6]

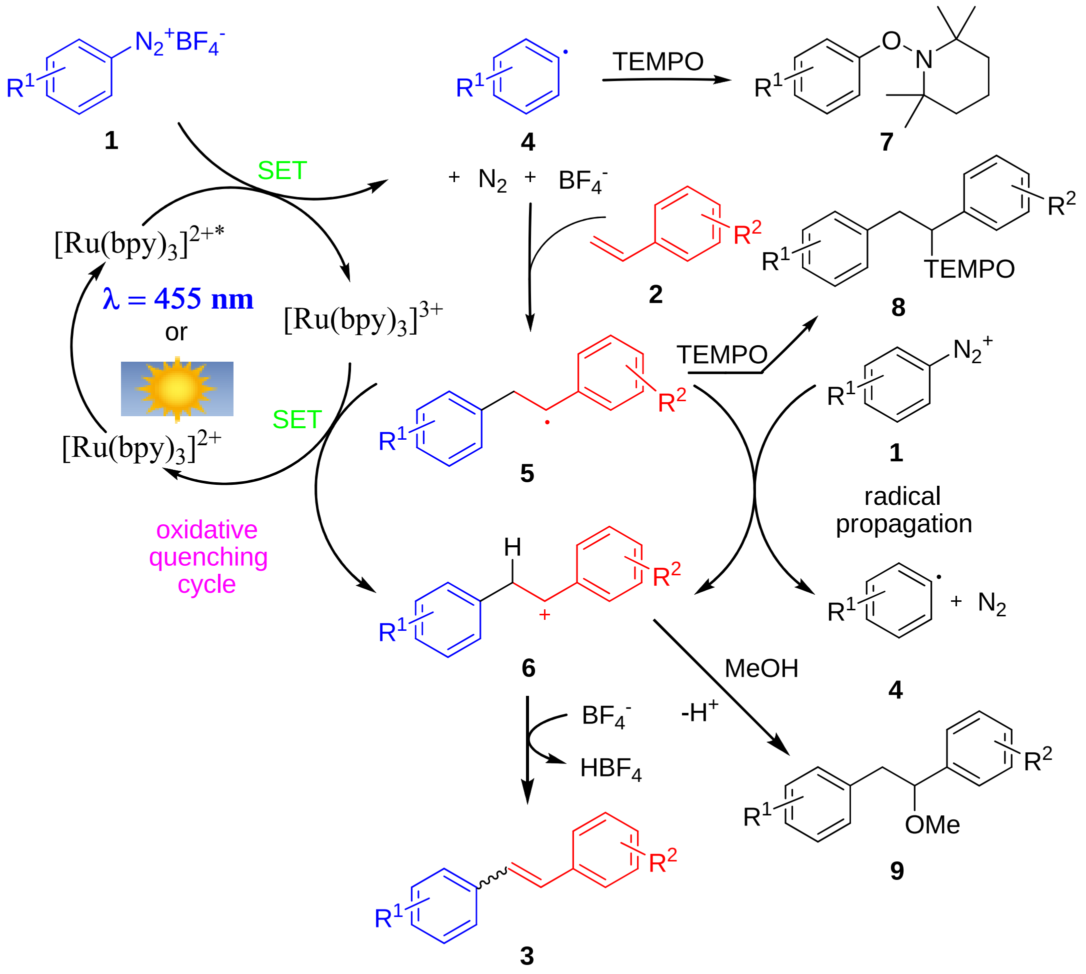

Scheme S-1. Mechanistic Model for the photoredox arylation of unsaturated compounds

The catalytic cycle starts with the excitation of the catalyst upon irradiation with blue light forming a $[Ru(bpy)_3]^{2+*}$ excited state that donates an electron to the aryl diazonium salt **1** which is known to act as an oxidative quencher for the excited state of the catalyst. The photocatalyst is oxidized forming a Ru(III)-species. The reduced form of the aryl diazonium salt is unstable and loses molecular nitrogen forming aryl radical **4**. The existence of **4** has been shown by trapping

the radical with TEMPO and the resulting adduct **7** has been detected. The highly reactive aryl radical attacks unsaturated compound **2** forming adduct radical **5** which is stabilized by its benzylic position. Again, a trapping experiment with TEMPO led to product **8** indicating the existance of a radical species. Intermediate radical **5** can be oxidized to the carbenium ion **6** either by re-donating an electron to the photocatalyst and simultaneously closing the catalytic cycle or by reducing another diazoinium salt molecule **1** initiating a radical chain mechanism. A control experiment that was performed by irradiating the mixture for ten minutes followed by stirring for another two hours in the dark showed that the radical chain must be very short or does even not occur since the yield did not increase after switching off the light. Starting from carbenium ion **6**, the product **3** is formed after deprotonation. The existence of **6** has been proved by a trapping experiment with MeOH as the nucleophile leading to product **9**.

In all, a vinylic H-atom has been replaced by an aryl residue. The utilization of BF_4^- as a non-nucleophilic counter-ion is important to avoid addition products of type **8**. However, the systematic addition of a nucleophile may be useful for the reaction because it is able to form a second bond in this type of reaction. Nevertheless, the nucleophile must not be too basic since the presence of strong bases leads to decomposition of the diazonium salts.

5. Experimental Data

Aryl diazonium tetrafluoroborates 1

Phenyl diazonium tetrafluoroborate **1a**

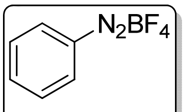

1a

^{1}H-NMR (400 MHz, DMSO-d_6): δ [ppm] = 7.98 (t, J = 6.9 Hz, 2H), 8.26 (t, J = 6.8 Hz, 1H), 8.66 (d, J = 7.4 Hz, 2H).
MS (ESI): *m/z* (%) = 105.0 (100) [M^+], 146.0 (96) [M^++MeCN].

4-Methoxyphenyl diazonium tetrafluoroborate **1b**

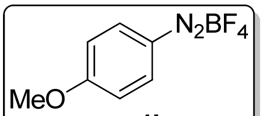

1b

^{1}H-NMR (400 MHz, DMSO-d_6): δ [ppm] = 4.04 (s, 3H), 7.46 – 7.51 (m, 2H), 8.59 – 8.64 (m, 2H).
MS (ESI): *m/z* (%) = 135.0 (100) [M^+], 175.9 (14) [M^++MeCN].

4-Methylphenyl diazonium tetrafluoroborate **1c**

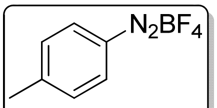

1c

^{1}H-NMR (400 MHz, DMSO-d_6): δ [ppm] = 2.57 (s, 3H), 7.79 (d, J = 8.4 Hz, 2H), 8.54 (d, J = 8.4 Hz, 2H).
MS (ESI): *m/z* (%) = 119.0 (100) [M^+], 160.0 (32) [M^++MeCN].

4-Nitrophenyl diazonium tetrafluoroborate **1d**

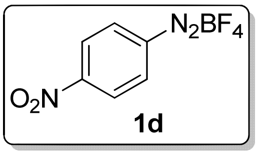

^{1}H-NMR (400 MHz, DMSO-d$_6$): δ [ppm] = 8.69 – 8.74 (m, 2H), 8.90 – 9.95 (m, 2H).

4-Chlorophenyl diazonium tetrafluoroborate **1e**

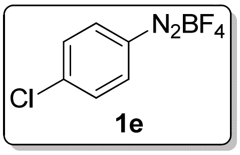

^{1}H-NMR (400 MHz, DMSO-d$_6$): δ [ppm] = 8.03 – 8.19 (m, 2H), 8.60 – 8.76 (m, 2H).
MS (ESI): *m/z* (%) = 138.9 (100) [M$^+$], 179.9 (88) [M$^+$+MeCN].

4-Bromophenyl diazonium tetrafluoroborate **1f**

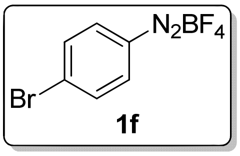

^{1}H-NMR (400 MHz, DMSO-d$_6$): δ [ppm] = 8.22 – 8.29 (m, 2H), 8.54 – 8.60 (m, 2H).
MS (ESI): *m/z* (%) = 184.8 (100) [M$^+$], 225.8 (80) [M$^+$+MeCN].

Stilbenes 3a – 3f

trans-stilbene **3a**

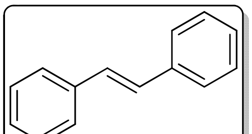

3a

^{1}H-NMR (300 MHz, $CDCl_3$): δ [ppm] = 7.17 (s, 2H), 7.28 – 7.36 (m, 2H), 7.37 – 7.46 (m, 4H), 7.53 – 7.61 (m, 4H).

^{13}C-NMR (75 MHz, $CDCl_3$): δ [ppm] = 126.7, 127.8, 128.8, 137.5.

(E)-1-Methoxy-4-styrylbenzene **3b**

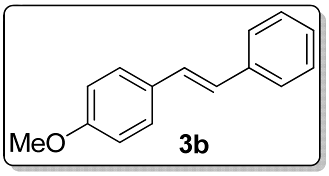

3b

^{1}H-NMR (300 MHz, DMSO-d_6): δ [ppm] = 3.77 (s, 3H), 6.95 (m, 2H), 7.09 (d, *J* = 16.5 Hz, 1H), 7.19 (d, *J* = 16.4 Hz, 1H), 7.24 (d, *J* = 7.5, 1H), 7.36 (t, *J* = 7.5 Hz, 2H), 7.55 (m, 4H).

MS (EI): *m/z* (%) = 210.1 (100) [M^+].

(E)-1-Methyl-4-styrylbenzene **3c**

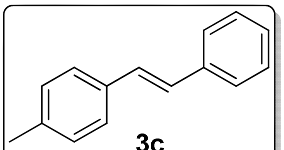

3c

^{1}H-NMR (300 MHz, $CDCl_3$): δ [ppm] = 2.38 (s, 3H), 7.05 (d, *J* = 16.5 Hz, 1H), 7.13 (d, *J* = 16.5 Hz, 1H), 7.19 (d, *J* = 8.0 Hz, 2H), 7.24 – 7.29 (m, 1H), 7.34 - 7.39 (m, 2H), 7.43 (d, *J* = 8.0 Hz, 2H), 7.52 (m, 2H)

^{13}C-NMR (75 MHz, $CDCl_3$): δ [ppm] = 21.4, 126.5, 126.6, 127.5, 127.8, 128.7, 128.8, 129.4, 129.5, 134.7, 137.7.

MS (EI): *m/z* (%) = 194.1 (100) [M^+], 179.1 (85) [M^+-CH_3·].

(E)-1-Nitro-4-styrylbenzene **3d**

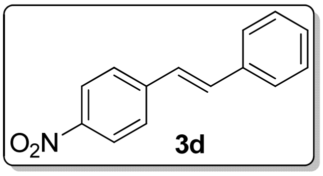

^{1}H-NMR (300 MHz, $CDCl_3$): δ [ppm] = 7.14 (d, *J* = 16.3 Hz, 1H), 7.28 (d, *J* = 16.3 Hz, 1H), 7.29 – 7.46 (m, 3H), 7.56 (m, 2H), 7.64 (m, 2H), 8.22 (m, 2H).
^{13}C-NMR (75 MHz, $CDCl_3$): δ [ppm] = 124.3, 126.4, 127.0, 127.2, 129.0, 129.1, 133.5, 136.3, 144.0,146.9.
MS (EI): *m/z* (%) = 178.1 (100) [M^{+}-HNO_2], 225.1 (86) [M^{+}].

(E)-1-Chloro-4-styrylbenzene **3e**

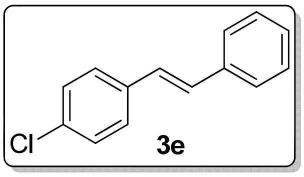

^{1}H-NMR (400 MHz, DMSO-d_6): δ [ppm] = 7.04 (d, *J* = 18.5 Hz, 1H), 7.10 (d, *J* = 18.5 Hz, 1H), 7.28 (t, *J* = 6.9 Hz, 1H), 7.33 (m, 2H), 7.37 (t, *J* = 6.9 Hz, 2H), 7.44 (m, 2H), 7.51 (d, *J* = 6.9 Hz, 2H).
MS (EI): *m/z* (%) = 179.1 (100) [M^{+}-Cl·], 199.1 (10) [M^{+}-CH_3·], 214.1 (70) [M^{+}].

(E)-1-Bromo-4-styrylbenzene **3f**

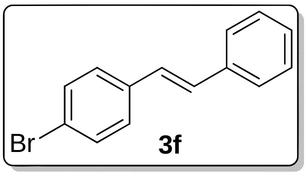

^{1}H-NMR (400 MHz, $CDCl_3$): δ [ppm] = 6.51 (d, *J* = 12.2 Hz, 1H), 6.64 (d, *J* = 12.2 Hz, 1H), 7.11 (d, *J* = 8.4 Hz, 2H), 7.23 (m, 5H), 7.34(d, *J* = 8.4 Hz, 2H).
MS (EI): *m/z* (%) = 179.1 (100) [M^{+}-Br·], 258.0 (40) [M^{+}].

Diphenylacetylenes 3g – 3h

1-Methoxy-4-(phenylethynyl)benzene **3g**

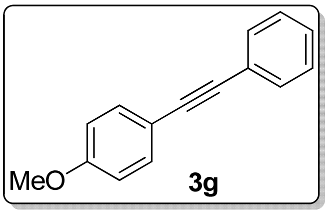

^{1}H-NMR (400 MHz, DMSO-d_6): δ [ppm] = 3.79 (s, 3H), 6.09 (m, 2H), 7.40 (m, 3H), 7.48 – 7.53 (m, 4H).

^{13}C-NMR (100 MHz, DMSO-d_6): δ [ppm] = 55.3, 87.9, 89.5, 114.2, 114.4, 122.7, 128.4, 128.7, 131.2, 132.9, 159.6.

MS (EI): *m/z* (%) = 193.1 (48) [M^{+}-CH_3·], 208.2 (100) [$M^{\cdot+}$].

1-Chloro-4-(phenylethynyl)benzene **3h**

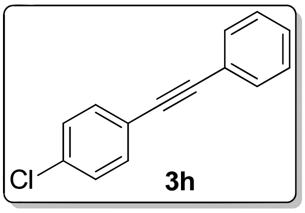

^{1}H-NMR (400 MHz, DMSO-d_6): δ [ppm] = 7.40 – 7.44 (m, 3H), 7.47 – 7.51 (m, 2H), 7.53 – 7.60 (m, 4H).

^{13}C-NMR (100 MHz, DMSO-d_6): δ [ppm] = 88.1, 90.3, 121.1, 122.0, 128.8, 128.9, 129.0, 131.4, 133.1, 133.5.

MS (EI): *m/z* (%) = 176.1 (35) [$M^{\cdot+}$-Cl·], 212.1 (100) [$M^{\cdot+}$].

Enones 3i – 3k

[1,1'-biphenyl]-2,5-dione **3i**

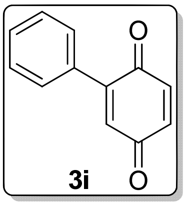

^{1}H-NMR (300 MHz, $CDCl_3$): δ [ppm] = 6.83 – 6.89 (m, 3H), 7.42 – 7.51 (m, 5H).

^{13}C-NMR (75 MHz, $CDCl_3$): δ [ppm] = 128.7, 129.4, 130.3, 130.7, 132.8, 132.9, 136.4, 137.2, 187.8, 188.8.

MS (EI): *m/z* (%) = 156.1 (58) [M^+-CO·], 184.1 (100) [M^+].

3-phenyl-2H-chromen-2-one **3k**

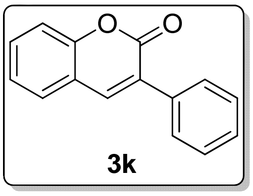

^{1}H-NMR (300 MHz, $CDCl_3$): δ [ppm] = 7.27 – 7.33 (m, 1H), 7.39 (m, 2H), 7.44 (m, 2H), 7.55 (m, 2H), 7.71 (m, 2H), 7.82 (s, 1H).

^{13}C-NMR (75 MHz, $CDCl_3$): δ [ppm] = 116.5, 119.7, 124.5, 127.9, 128.4, 128.5, 128.6, 128.9, 131.4, 134.7, 139.9, 153.6, 160.6.

MS (EI): *m/z* (%) = 194.1 (78) [M^+-CO·], 222.1 (100) [M^+].

Trapped Intermediates 7 – 9

2,2,6,6-tetramethyl-1-phenoxypiperidine **7** (R_1 = H)

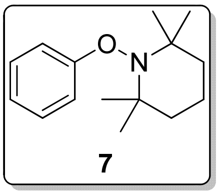

MS (CI): *m/z* (%) = 234.2 [MH^+].

1-(1,2-diphenylethoxy)-2,2,6,6-tetramethylpiperidine **8** (R_1 = R_2 H)

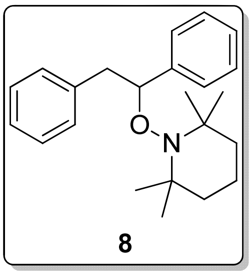

MS (ESI): *m/z* (%) = 338.2 [MH^+].

(1-methoxyethane-1,2-diyl)dibenzene **9** (R_1 = R_2 = H)

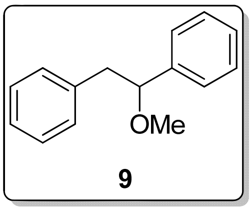

^{1}H-NMR (300 MHz, $CDCl_3$): δ [ppm] = 2.89 (dd, ^2J = 13.8 Hz, ^3J = 5.8 Hz, 1H), 3.10 (dd, ^2J = 13.9 Hz, ^3J = 6.3 Hz, 1H), 3.19 (s, 3H), 4.33 (dd, ^3J = 6.5 Hz, ^3J = 5.9 Hz, 1H), 7.15 – 7.35 (m, 10 H).

^{13}C-NMR (75 MHz, $CDCl_3$): δ [ppm] = 44.9, 56.9, 85.2, 126.3, 126.9, 127.7, 128.2, 128.4, 129.6, 138.6, 141,8.

MS (EI): *m/z* (%) = 77.1 (18) [$C_6H_5^+$], 91.1 (14) [$C_7H_7^+$], 121.1 (100) [M^+-$C_7H_7^+$], 181.1 (4) [M^+-CH_3O·], 212.1 (1) [M^+].

6. References

[1] K. Schwetlick, *Organikum* **2009,** Wiley-VCH.

[2] P. Hanson, J. R. Jones, A. B. Taylor, P. H. Walton, A. W. Timms, *Journal of the Chemical Society, Perkin Transactions 2* **2002**, 1135-1150.

[3] H. Cano-Yelo, A. Deronzier, *Tetrahedron Letters* **1984**, *25*, 5517-5520.

[4] C. Galli, *Chem Rev* **1988**, *88*, 765-792

[5] D. Kalyani, K. B. McMurtrey, S. R. Neufeldt, M. S. Sanford, *J. Am. Chem. Soc.* **2011**, *133*, 18566-18569.

[6] F. Teplý, *Collection of Czechoslovak Chemical Communications* **2011**, *76*, 859-917.
